# Integrating Caregivers in Health Care Delivery Models

**DOI:** 10.1093/geroni/igaf122.1190

**Published:** 2025-12-31

**Authors:** Yadira Montoya

**Affiliations:** National Alliance for Caregiving, Washington, District of Columbia, United States

## Abstract

NAC’s presentation will outline strategies for integrating family caregivers into healthcare delivery models, with emphasis on implementing reimbursable Caregiver Training Services (CTS). We will highlight family caregivers’ roles in cancer care, present a workflow for CTS integration, and provide a six-point checklist for program development. The presentation will showcase case studies from Northwell Health and RUSH University Medical Center, demonstrating real-world considerations for CTS implementation in geriatric and serious illness care, complemented by practical tools and evidence-based resources for effective caregiver integration.

